# Do the Levels of Maternal Plasma Trace Elements Affect Fetal Nuchal Translucency Thickness?

**DOI:** 10.1371/journal.pone.0138145

**Published:** 2015-09-14

**Authors:** Kai-Wei Liao, Ming-Song Tsai, Chia-Huang Chang, Ling-Chu Chien, I-Fang Mao, Yen-An Tsai, Mei-Lien Chen

**Affiliations:** 1 Institute of Environmental and Occupational Health Sciences, School of Medicine, National Yang Ming University, Taipei, Taiwan; 2 Department of Obstetrics and Gynecology, Cathay General Hospital, Taipei, Taiwan; 3 School of Medicine, Fu Jen Catholic University, Taipei, Taiwan; 4 School of Medicine, Taipei Medical University, Taipei, Taiwan; 5 School of Public Health, Taipei Medical University, Taipei, Taiwan; 6 Department of Occupational Safety and Health, Chung Shan Medical University, Taichung, Taiwan; University of Rennes-1, FRANCE

## Abstract

**Objective:**

Fetal nuchal translucency (NT) thickness is an important marker for prenatal screening; however, studies focusing on the correlation between maternal trace element levels and NT thickness are limited. The aim of this study was to evaluate maternal trace element levels during the first trimester and to investigate the association between maternal trace element levels and fetal NT thickness.

**Methods:**

In total, 113 samples were obtained from singleton pregnant women. Maternal plasma samples were collected in the first trimester of gestation. Plasma trace element levels were measured using Inductively Coupled Plasma Mass Spectrometry (ICP-MS). Nuchal translucency thickness was measured using ultrasonography at 10–14 weeks of gestation.

**Results:**

We found that maternal plasma potassium (K) levels had a significant negative correlation with both NT (r = -0.230, *p* < 0.05) and NT Multiples of the Median (NT MoM) (r = -0.206, *p* < 0.05). After adjustment for potential confounders, log-transformed maternal plasma potassium levels in the first trimester were significantly associated with fetal NT (NT MoM: β = -0.68, *p* < 0.05; NT: β = -1.20, *p* < 0.01). Although not statistically significant, the As, Hg and Pb levels in maternal plasma were positively correlated with NT, and the Mg, Cu, Zn, Na and Ca levels were negatively correlated with NT.

**Conclusion:**

Maternal plasma K levels during the first trimester appeared to be associated with NT thickness. The essential elements tended to decrease NT thickness, and non-essential elements tended to increase it.

## Introduction

Trace elements, both essential and non-essential, play an important role in the maintenance of fetal development. Optimal amounts of essential trace elements, such as copper (Cu), potassium (K), calcium (Ca), sodium (Na), magnesium (Mg) and zinc (Zn), are crucial for the human body, but amounts exceeding the range can be toxic. Pregnant women may be susceptible to trace element deficiencies because of alterations in nutritional intake throughout the course of pregnancy [1]. Although trace elements are essential nutritional components for humans, there is limited knowledge about their specific roles, especially during the fetal period. Arsenic (As), mercury (Hg) and lead (Pb) are known as non-essential metals. These metals have been extensively studied in recent decades in response to concerns that they may have an impact on pregnancy outcomes and have adverse developmental effects [2,3,4]. During pregnancy, the placenta acts as a barrier by allowing nutrients and oxygen to pass to the fetus while preventing toxic substances from crossing through [5,6,7]. However, previous in vivo and in vitro studies have reported that As, Pb, and Hg can easily cross the placenta and may accumulate in the fetus [8,9].

Nuchal translucency (NT) refers to the subcutaneous space in the fetal neck and is visible with ultrasound imaging in the first trimester [10,11,12]. Nuchal translucency thickness increases with increasing gestational age and fetal crown-rump length [11,13,14,15]. Currently, NT measurements are offered in most countries as part of the first trimester screening for Down syndrome, and this measurement has been proven an effective method for chromosomal anomaly screening [13,16,17,18]. Fetuses with increased NT thickness in the first trimester have been reported to be at risk for chromosomal abnormalities, genetic syndromes, congenital heart defects, structural abnormalities, intrauterine infection, neurodevelopmental delay, and fetal demise [19,20,21,22,23,24,25,26,27]. Although the pathophysiology responsible for the increased NT thickness remains unclear, possible explanations for the accumulation of fluid include cardiac failure associated with heart defects, venous congestion in the head and neck, an altered composition of the extracellular matrix, the abnormal or delayed development of the lymphatic system, a failure of lymphatic drainage resulting from impaired fetal movements related to various neuromuscular disorders, fetal anaemia or hypoproteinamia, and congenital infection [16,28,29,30,31,32,33]. Nuchal translucency thickness is an important marker for prenatal screening, but no studies of the correlation between maternal trace element levels and NT thickness have been reported. Furthermore, only limited studies have analyzed the factors accounting for the false-negative results of Down syndrome screening. It remains unclear whether maternal plasma trace element levels affect NT thickness and potentially lead to increased false-negative or false-positive rates in Down syndrome screening.

The aim of this study was to evaluate the maternal plasma trace element levels during the first trimester and to investigate the association between these levels and fetal NT thickness. Given the identification of such associations, obstetricians should be aware of the impact of maternal plasma trace elements on first trimester Down syndrome screening.

## Materials and Methods

### Study subjects

Pregnant women were recruited from a single institution in northern Taiwan. After informed consent was obtained, pregnant women who had undergone Down syndrome screening during the first trimester of gestation were invited to participate in the study between March and December 2010. In total, 113 pregnant women participated in this study, and 113 singleton births occurred. Down syndrome screening involves measuring the fetal NT thickness and the maternal serum levels of free β-hCG and pregnancy-associated plasma protein A (PAPP-A) during gestational weeks 10–14. Among the participating pregnant women, 8 pregnant women were screened at 10 weeks of gestation, 55 pregnant women at 11 weeks, 43 pregnant women at 12 weeks, 6 pregnant women at 13 weeks and 1 pregnant woman at 14 weeks. Maternal plasma samples were collected at the same time.

Each pregnant woman was asked questionnaire consisting of information on sociodemographic characteristics (age, weight, height, and education), lifestyle (smoking, drinking, exercise, supplement use, and medication) and dietary consumption (such as meat, vegetable, fruit, tea and coffee consumption). Personal and family disease histories, allergy histories, and reproductive histories were acquired from the hospital’s information system.

### Ethics statement

The research protocol was approved by the Institutional Review Board of Cathay General Hospital, Taipei, Taiwan (Institutional Review Board number: CT9787, approved on 2009.02.18). Written informed consent was obtained from all participants.

### Plasma trace element measurements

Maternal plasma samples were collected at the first-trimester Down syndrome screening. A total of 113 plasma samples were obtained. After the samples were collected, they were immediately chilled and shipped in batches to National Yang-Ming University. The samples were stored at -80°C until analysis.

The plasma samples were digested with 65% nitric acid. After digestion, the plasma trace elements were analyzed using Inductively Coupled Plasma Mass Spectrometry (ICP-MS) (Thermo Scientific X-series II, Bremen, Germany). The analyzed elements included As, Pb, Cd, Mg, Cu, Zn, Na, K and Ca. The recovery rate of the plasma trace elements content ranged from 90–106%. The correlation coefficients (R^2^) of the standard curves were greater than 0.995. Each specimen was calibrated and adjusted using a blank sample. The results are reported as the mean of two replicate measurements. The limit of detection divided by 2 was utilized to incorporate samples below the detection limit because the data were highly skewed [34]. The accuracy of the method was evaluated using a human serum certified reference material (Seronorm™ L-2 Trace Elements ref. MI0181 SERO AS, Billingstad, Norway).

### Fetal NT measurement

Fetal NT thickness was measured at 10–14 weeks of gestation by a gynecologist and three trained sonographers using an Acuson 128 ultrasonography unit (Mountain View, CA, USA) according to the criteria published by the Fetal Medicine Foundation in the UK [35]. The measurement was obtained using a transabdominal approach in the sagittal plane with the fetus occupying three-quarters of the image. During the NT measurement, fetal scanning was performed thoroughly to determine whether there were any fetal structural abnormalities. The NT values were divided by their respective day-specific median levels to determine the Multiples of the Median (MoM).

### Statistical analysis

The biochemical parameters are presented as the mean, and the plasma trace element levels are presented as the geometric mean. The correlations between fetal NT and trace element levels were determined using Spearman's correlation test. Because of the skewed distribution, the plasma trace element levels were assessed using the Kolmogorov-Smirnov test, and the plasma trace element levels underwent a natural log transformation. Potential confounders associated with birth outcomes were considered according to both maternal and paternal factors. Multiple linear regression models were used to describe the relationships between fetal NT as a dependent quantitative variable and maternal plasma trace element levels. The potential confounders associated with birth outcomes were considered according to maternal and paternal factors, including maternal age, gestational weeks, pre-pregnancy BMI, supplement use and medication, and statistical considerations were applied when the covariates were significant in the univariate models. A threshold of 0.1 was determined for covariates selection. The Kruskal-Wallis test was performed to compare the NT and NT MoM grouped by the tertiles of the plasma K concentration. All statistical analyses were performed using the Statistical Package for the Social Sciences version 17.0 (SPSS Inc., Chicago, IL). Statistical significance was defined as *p*<0.05.

## Results

Only singleton pregnant women were analyzed in this study. A total of 113 women participated in the study. The sociodemographic characteristics are shown in Table 1. The mean (standard deviation (SD)) age of the pregnant women was 31 (3.1) years old. Overall, 55 (50.5%) of the pregnant women were primiparous. Eight fetuses were screened at 10 weeks of gestation, 55 fetuses at 11 weeks, 43 fetuses at 12 weeks, 6 fetuses at 13 weeks and 1 fetus at 14 weeks. Approximately 85% of the pregnant women had graduated from college or had higher levels of education. The mean maternal Body Mass Index (BMI) in the first trimester was 21.65 (2.96). Seventy-five (66.4%) of the women reported using supplements during pregnancy. There were two (1.7%) pregnant women who smoked and one (0.9%) who drank alcohol during her pregnancy (Table 1).

**Table 1:** Sociodemographic characteristics of the study subjects.

	N	%	Mean (SD)
**Maternal age**			30.92(3.09)
≦31	60	53.1	
32–34	42	37.2	
≧35	11	9.7	
**Primipara**			
Yes	55	50.5	
No	54	49.5	
**Gestational week**			
≦11	63	55.8	
12	43	38.1	
≧13	7	6.1	
**Maternal education**			
Senior high school	18	15.9	
College	16	14.2	
University	60	53.1	
Graduate school	19	16.8	
**Maternal BMI**			21.65(2.96)
Underweight (<18.5)	22	19.5	
Normal weight (18.5–24.9)	79	69.9	
Overweight (25.0–29.9)	9	8.0	
Obese (>30)	3	2.6	
**Supplement use**			
No	38	33.6	
Yes	75	66.4	
**Medication**			
No	105	92.9	
Yes	8	7.1	
**Exercise**			
Rare	111	98.2	
Often	2	1.8	
**Smoking**			
No	111	98.2	
Yes	2	1.8	
**Drinking**			
No	112	99.1	
Yes	1	0.9	

Table 2 presents the mothers’ biochemical parameters and plasma trace element levels. The data presented using the NT MoM approach appeared reasonable [36]. The median NT thicknesses (NT MoM) were 0.95 mm (0.88), 1.3 mm (1.14), 1.4 mm (1.04), 1.65 mm (1.19), and 1.4 mm (0.76) at 10, 11, 12, 13 and 14 weeks of gestation, respectively. The geometric mean plasma trace element levels of As, Hg, Pb, Zn, Cu, Mg, Ca, K and Na were 0.69 μg/L, 1.67 μg/L, 0.048 μg/L, 647.2 μg/L, 1.10 mg/L, 22.42 mg/L, 96.18 mg/L, 187.6 mg/L and 2973.5 mg/L, respectively (Table 2).

**Table 2:** Biochemical parameters and plasma trace element levels.

	NT thickness (mm)		NT MoM	
Week	Median	(range)	Median	(range)
10 (n = 8)	0.95	(0.70–1.40)	0.88	(0.65–1.21)
11 (n = 55)	1.30	(0.90–2.30)	1.14	(0.77–1.99)
12 (n = 43)	1.40	(0.90–2.40)	1.04	(0.70–1.73)
13 (n = 6)	1.65	(1.40–2.20)	1.19	(0.86–1.43)
14 (n = 1)	1.20	-	0.76	-
**Plasma**	**Geometric mean (G.S.D.)**			
*Unit*:*μg/L*				
As	0.69		(1.92)	
Hg	1.67		(4.24)	
Pb	0.048		(5.92)	
*Unit*:*mg/L*				
Zn	0.65		(2.47)	
Cu	1.10		(1.65)	
Mg	22.42		(1.19)	
Ca	96.18		(1.21)	
K	187.6		(1.17)	
Na	2973.5		(1.07)	

NT MoM: Nuchal translucency multiples of the median.

G.S.D: Geometric standard deviation

The spearman correlation coefficients between NT and plasma trace element levels are presented in Table 3. The results showed that plasma K levels were negatively correlated with NT (r = -0.230, *p*<0.05) and NT MoM (r = -0.206, *p*<0.05). Although the results were not statistically significant, As, Hg and Pb were positively correlated with NT and NT MoM. Mg, Cu, Zn, Na and Ca levels were negatively correlated with NT MoM (Table 3).

**Table 3:** Spearman correlation coefficients between plasma trace element levels and fetal nuchal translucency.

	As	Hg	Pb	Zn	Cu	Mg	Ca	K	Na
*Unit*:	μg/L			mg/L					
NT (mm)	0.097	0.086	0.103	0.001	0.028	0.017	-0.024	-0.230*	-0.105
NT MoM	0.096	0.093	0.081	-0.091	-0.036	-0.014	0.051	-0.206*	-0.121

* *p*<0.05

To explore the association between plasma trace element levels and fetal NT, the multivariate model was adjusted for maternal age, pre-pregnancy BMI, gestational weeks, and medication and supplement use. After adjustment for potential confounders, the log transformed maternal levels of maternal plasma essential elements (Zn, Cu, Ca, Mg, K and Na) were negatively associated with fetal NT and NT MoM. The levels of non-essential elements (Pb, Hg and As) were positively associated with fetal NT and NT MoM. Plasma K levels were significantly associated with fetal NT thickness (NT MoM: β = -0.68, *p*<0.05; NT: β = -1.20, *p*<0.01) (Table 4).

**Table 4:** Multiple linear regression models of fetal NT and plasma trace element levels.

	NT_MoM ^a^	NT ^b^
	β (95% CI)	β (95% CI)
Log transformed		
*Unit*:*μg/L*		
**As**	0.098 (-0.06, 0.25)	0.149 (-0.07, 0.36)
**Hg**	0.019 (-0.06, 0.09)	0.005 (-0.97, 0.11)
**Pb**	0.011 (-0.05, 0.07)	0.022 (-0.06, 0.10)
*Unit*:*mg/L*		
**Zn**	-0.039 (-0.15, 0.07)	-0.025 (-0.18, 0.13)
**Cu**	-0.121 (-0.33, 0.09)	-0.104 (-0.39, 0.18)
**Mg**	-0.259 (-0.84, 0.33)	-0.288 (-1.09, 0.52)
**Ca**	-0.024 (-0.56, 0.51)	-0.166 (-0.89, 0.56)
**K**	-0.684 (-1.32, -0.05)*	-1.204 (-2.06, -0.34)**
**Na**	-0.707 (-2.41, 1.00)	-0.363 (-2.72, 2.00)	

*p<0.05

** *p*<0.01

^a^ Model adjusted for maternal age, pre-pregnancy BMI, medication and supplement use.

^b^ Model adjusted for maternal age, pre-pregnancy BMI, gestational weeks, medication and supplement use.

Furthermore, we divided the pregnant women into two groups (multiparas and primiparas) to evaluate the association between maternal plasma trace element levels and fetal NT thickness according parity. After log transformation, the level of maternal plasma K was negatively associated with fetal NT in primiparous group (NT: β = -1.764, *p*<0.05). Plasma Pb level was positively associated with fetal NT in multiparous group (NT: β = 0.107, *p*<0.05; Table 5).

**Table 5:** Multiple linear regression models of fetal NT and plasma trace element levels in primiparas and multiparas.

	Multiparas (n = 54)		Primiparas (n = 55)	
	NT_MoM ^a^	NT ^b^	NT_MoM ^a^	NT ^b^
Log transformed	β (95% CI)	β (95% CI)	β (95% CI)	β (95% CI)
*Unit*:*μg/L*				
**As**	-0.074 (-0.281, 0.134)	-0.105 (-0.392, 0.181)	0.148 (-0.112, 0.407)	0.203 (-0.175, 0.580)
**Hg**	-0.051 (-0.140, 0.038)	-0.108 (-0.231, 0.015)	0.079 (-0.045, 0.203)	0.110 (-0.069, 0.289)
**Pb**	0.066 (-0.009, 0.141)	0.107 (0.003, 0.212) *	-0.041 (-0.131, 0.048)	-0.065 (-0.194, 0.065)
*Unit*:*mg/L*				
**Zn**	-0.029 (-0.182, 0.125)	0.013 (-0.204, 0.230)	-0.065 (-0.221, 0.091)	-0.044 (-0.293, 0.205)
**Cu**	0.022 (-0.231, 0.274)	0.129 (-0.227, 0.485)	-0.155 (-0.519, 0.209)	-0.225 (-0.748, 0.298)
**Mg**	0.016 (-0.648, 0.681)	0.008 (-0.934, 0.950)	-0.328 (-1.436, 0.779)	0.192 (-1.415, 1.798)
**Ca**	0.598 (-0.147, 1.333)	0.503 (-0.568, 1.574)	-0.204 (-1.033, 0.625)	-0.239 (-1.436, 0.959)
**K**	-0.239 (-1.032, 0.554)	-0.453 (-1.579, 0.673)	-0.859 (-2.055, 0.337)	-1.764 (-3.453, -0.076) *
**Na**	-0.253 (-2.360, 1.854)	-0.033 (-3.040, 2.975)	-1.919 (-4.900, 1.062)	0.028 (-4.180, 4.237)

*p<0.05

** *p*<0.01

^a^ Model adjusted for maternal age, pre-pregnancy BMI, medication and supplement use.

^b^ Model adjusted for maternal age, pre-pregnancy BMI, gestational weeks, medication and supplement use.

**Fig 1:**
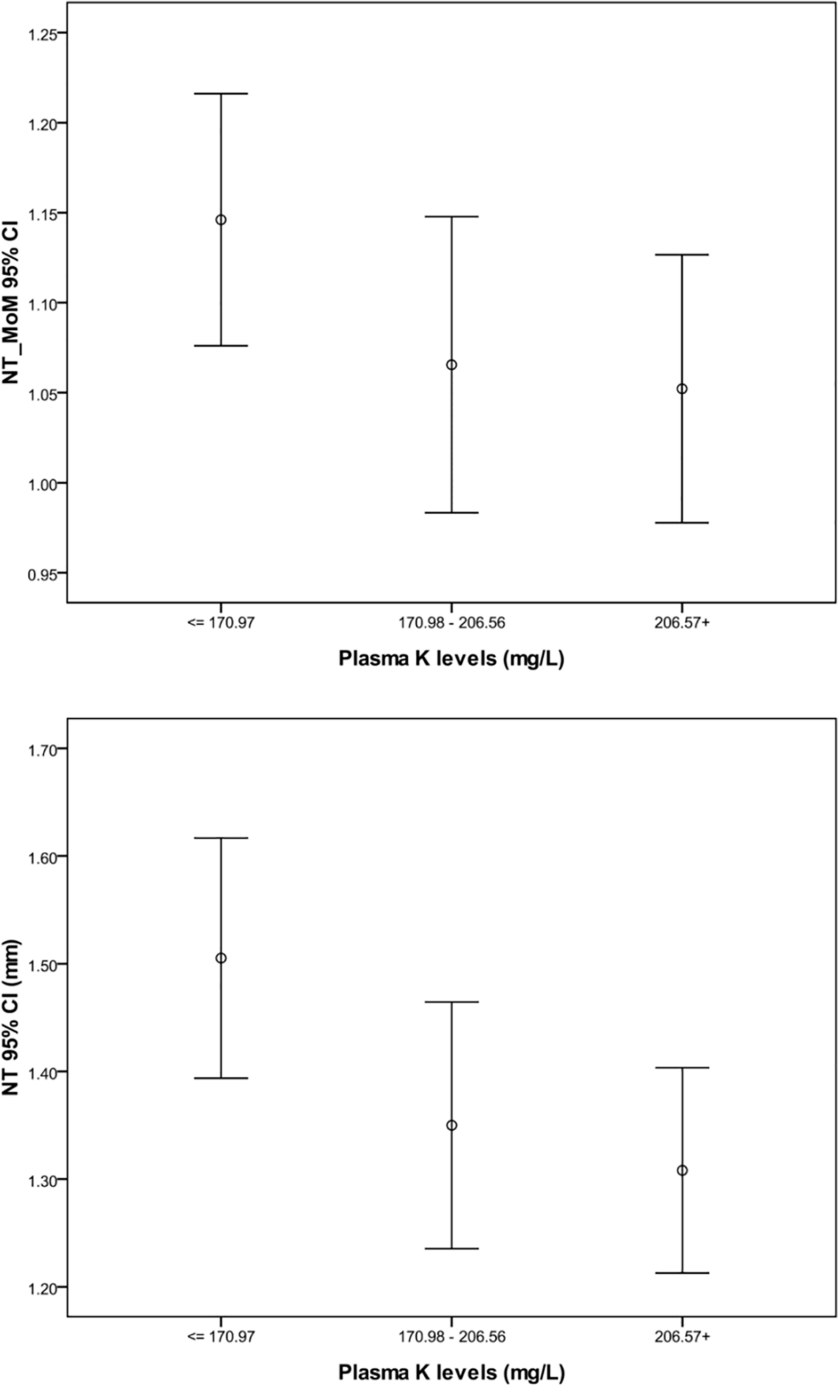
Fetal NT and NT MoM according to maternal plasma potassium levels.

To clarify the association between plasma K levels and fetal NT, we categorized the fetus into three groups according to their maternal plasma K levels. The maternal plasma K levels were grouped into tertiles: first tertile, less than 170.97 mg/L; second tertile: 170.98–206.56 mg/L; and third tertile, greater than 206.57 mg/L. The NT thicknesses (mean±SD) were 1.51±0.34 mm, 1.35±0.35 mm and 1.31±0.29 mm in the first, second, and third tertiles, respectively. The respective NT MoM values (mean±SD) were 1.15±0.21, 1.07±0.25 and 1.05±0.23. Using the Kruskal-Wallis test, we determined that the fetal NT and maternal plasma K levels differed significantly among the three groups (Fig 1). Low K concentrations were correlated with greater NT thickness.

## Discussion

The findings of this study suggest that a relationship exists between maternal plasma trace element levels and fetal NT thickness during the first trimester. The essential elements tended to decrease NT thickness, and non-essential elements tended to increase it. Maternal plasma K levels during the first trimester appeared to be associated with NT thickness.

Pregnant women must retain additional fluid and electrolytes to meet both their needs and those of the growing fetus [36]. However, nausea and vomiting in pregnancy generally begin between 4 and 6 weeks of gestation and peak between 8 and 12 weeks [37]. These common symptoms of pregnancy affect approximately 70–85% of pregnant women [38]. Severe vomiting can cause imbalances in electrolytes and trace elements. Potassium is an electrolyte that maintains water and electrolyte balance. Most K is stored as an intracellular cation in the fetus space and in the placenta and uterus [39]. Only 2% of internal K is located in the extracellular fluid [39]. Potassium levels in the extracellular fluid are regulated by mechanisms that control the internal distribution between the intracellular and extracellular compartments and the external balance between intake and output [40]. The fetus and newborn must conserve K for growth [40]. In fetal life, this need is met by the active transport of K across the placenta from mother to fetus [41]. However, the mechanism of action of maternal plasma-derived K in the fetus is not fully understood. A previous study reported that the K concentration in the saliva of children with Down syndrome (21.02±2.21 mEq/L) was significantly lower than that of mentally retarded children without Down syndrome (23.27±3.98 mEq/L) and that of normal children (23.58±2.93 mEq/L) [42]. In an ATPase activity study, Na and K concentrations were studied in the platelets of normal subjects and patients with Down syndrome; the results showed that the patients’ platelet K content was significantly decreased (13.12±0.48 μg per 10^9^ platelets vs. 19.78±0.72 μg per 10^9^ platelets; *p*<0.001) [43]. The normal range for plasma K levels is narrow, from 3.5 to 5.5 mEq/L. In our study, the mean plasma K level among the pregnant women was 4.87 mEq/L. After adjustment for potential confounders, a consistent negative association was found between maternal essential trace element levels and fetal NT; this association was statistically significant for plasma K (β = -1.204, *p*<0.01).

The retention of fluid and electrolytes begins immediately when a woman becomes pregnant [44]. After the implantation of the embryo, maternal fluid retention and the balance of Na and K become important. The fetus relies on maternal resources to acquire minerals that are indispensable for maintaining optimal fetal mineral levels and homeostasis [45]. Sodium and potassium uptake by the fetus is critical for growth and development but must be carefully controlled to maintain the correct osmotic and fluid balance in the fetal compartment [46]. However, the relationship between maternal fluid retention and NT thickness is unclear.

During pregnancy, parity seems to play an important role. In this study, there were no significant differences in fetal NT thickness between the primiparous and multiparous pregnant women. In the present study, 55 of the pregnant women were primiparous and 54 were multiparous. After adjustment for potential confounders, the NT thickness was associated with maternal plasma K levels in the primiparous pregnant women (NT: β = -1.764, *p*<0.05), and maternal plasma Pb was positively associated with NT thickness in multiparous pregnant women (NT: β = 0.107, *p*<0.05). A previous study reported that parity may influence placental efficiency of and fetal growth [47]. No study has mentioned the correlation between parity and NT thickness; however, the present study found that plasma K levels are associated with NT thickness in primiparas. The mechanism of interaction between parity and maternal plasma trace elements on fetal NT thickness needs to be further explored.

Nuchal translucency thickness increases progressively between 10 and 14 gestational weeks [13]. After 14 weeks of gestation, fetal NT thickness steadily decreases and then disappears [48]. In our study, NT thickness was measured between 11 and 12 weeks (94%) for most of the fetuses; only one (0.9%) fetus was measured at 14 weeks. The median fetal NT thickness in this study was 1.4 mm, with a range of 0.7 to 2.4 mm. A previous study reported that the NT thickness ranged from 1.2 mm to 1.9 mm in Japan [49]. In China, one study reported a mean NT thickness of 1.7 mm [50]. In America, the fetuses have a normal reported NT thickness range of 0.7 to 2.4 mm [51]. In a study performed in Serbia, the mean fetal NT thickness was 1.92 mm [52]. The fetal NT thickness in our study was similar to that in other countries.

The physiological basis of increased fetal NT thickness during the first trimester of pregnancy is not understood. One possible cause is a congenital heart defect, but it is difficult to explain the exact mechanism related to different types of congenital heart defects, each of which has its own corresponding haemodynamics [16,28,29,30,31]. Another possibility is the accumulation of fluid associated with overperfusion to protect fetus neural structure development [32]. Between 9 and 12 weeks of gestation, the placenta enters a stage of rapid growth with increased circulating blood volume in conjunction with a parallel type of fetal circulation [53]. During the early stages of central nervous system development, the neural structure and brain must be protected by overperfusion. In this study, we found a positive correlation between maternal plasma levels of non-essential metals and NT thickness. Although there were no statistically significant correlations found for these elements, the finding might speculate that the protective function of overperfusion in response to heavy metal exposure.

Many studies have reported associations between NT thickness, pregnancy outcomes, congenital heart disease, neurodevelopment and Down syndrome, but studies of false-negative screening results are limited [26,54,55,56,57]. Nuchal translucency measurements alone can identify approximately 60% of Down syndrome cases in the first trimester with a 5% false-positive rate [58]. A previous study in Finland reported that NT measurements were the most powerful discriminating factor in false-negative screening results and accounted for 37.2% of the results [59]. In this case, in the absence of a structural abnormality, most of false-negative cases will be born. One study mentioned that parents who receive a false-negative result may be angrier upon their child’s birth compare with parents who did not undergo screening [60]. In this study, we found that NT was negatively correlated with maternal plasma K levels, suggesting a possible factor that may increase the false-negative rate. Pregnant women with high K levels will exhibit decreased fetal NT thickness. Approximately 75% of Down syndrome fetuses have increased NT thickness [61]. The estimated detection rate for Down syndrome using a combination of maternal age, fetal NT thickness and maternal serum PAPP-A and free β-hCG levels is approximately 90%, with a screen-positive rate of 5%. The false-positive rate of Down syndrome screening is low, but the false-negative rate could be affected by high maternal plasma K levels.

After adjusting for potential confounders, maternal plasma K was correlated with fetal NT thickness. Potential confounders were identified based on previous studies or because the factor could be considered biologically plausible (e.g., maternal age, pre-pregnancy BMI and medication) or correlated (p<0.1) with plasma K levels and fetal NT thickness (e.g., gestational weeks and supplement use). In a previous study involving 100,311 pregnancies, the median increased from 1.2 mm at 11 weeks to 1.9mm at 13^+6^ weeks; therefore, it is essential to take gestational age into consideration when determining whether NT thickness is increased in a given fetus [18]. It is also well known that maternal age is associated with subfertility, chromosomal abnormalities and prenatal adverse outcomes [62,63,64,65]. One study also found that the presence of nausea and vomiting was associated with an older maternal age and increased BMI [66]. Severe vomiting may cause imbalances of electrolytes and bodily trace element levels. A previous study also indicated that maternal serum electrolyte levels were related to maternal weight gain [67]. In 2009, the Institute of Medicine highlighted both pre-pregnancy BMI and excess gestational weight gain as significant contributors to infant development [68]. A previous study identified correlations between fetal NT thickness and maternal BMI, smoking and ethnicity [69]. Pregnancy is accompanied by substantial physiological changes. The need for K support is increased, particularly during the first trimester of pregnancy [70]. The effects of dietary supplementation on a developing fetus are largely unknown. However, there are several dietary supplements (e.g., folic acid and iron) that may be beneficial if taken during pregnancy [71]. Several studies have examined whether micronutrient supplementation improves birth weight and reduce early infant mortality [65,72,73]. In the present study, approximately 67% of the women reported using supplements during their pregnancy. Supplement use may affect the body’s trace element levels. Medications are also important during pregnancy. A previous study reported that over-the-counter medications are used by most pregnant women, and that specific medications have been linked to specific birth defects [74]. Hendrick et al. (2003) reported that the use of high doses of medication throughout pregnancy may be associated with the risk of low birth weight [75]. Futhermore, specific drugs are known to affect plasma K levels [76]. In this study, eight women (7.1%) reported taking medications during pregnancy. Among these eight women, there were no differences in either fetal NT thickness (NT: 1.39 mm vs. 1.40 mm, *p* = 0.955; NT MoM: 1.09 vs. 1.07, *p* = 0.666) or plasma K levels (4.88 mEq/L vs. 4.63 mEq/L, *p* = 0.359) between the no-medication-use group and the medication-use group. Medication may be a modifying variable with respect to fetal NT thickness; therefore, it was included in the regression model. A previous study determined that the mechanisms underlying K and insulin uptake are linked; therefore, hypokalemia may induce impaired glucose tolerance [70]. DeFronzo et al. (1980) observed a relationship between decreased plasma K levels and both insulin levels and glucose intake [77]. However, Cohen et al. (1991) and Nguyen et al. (2011) observed that insulin exerts independent effects on both glucose and K uptake [78,79]. In the present study, only three women developed Gestational Diabetes Mellitus (GDM) during the second trimester. There were no differences between the women with GDM and the women with normoglycemia with respect to plasma K levels and fetal NT thickness. None of the fetuses in this study exhibited either chromosomal abnormalities or birth defects. Considering causal inferences, the potential confounders of maternal age, pre-pregnancy BMI, gestational weeks and medication and supplement use were controlled. We are aware that this study included relatively few subjects. However, the results were statistically significant.

This study has certain limitations. First, without fetuses with chromosomal abnormalities and birth defects, false-negative results were hard to establish. Second, based on the limited sample size, NT was significantly associated with maternal plasma K levels. A larger study is needed to confirm the findings. Third, we did not enroll the women until they were pregnant; therefore, we could not determine the temporal variation in plasma K levels among the pregnant women before and after pregnancy. In a previous study, the value recorded for the first trimester was 4.25 mmol/L, which increased to 5.83 mmol/L in the second trimester and to 5.95 mmol/L in the third trimester [80]. Maternal K levels were increased during pregnancy. In a German study, the serum-K levels of pregnant women after delivery were significantly increased compared with those of non-pregnant women [81]. In our study, the mean for maternal plasma K levels was 4.87 mmol/L. Compared with the general population, the pregnant women’s K levels was higher. Additional studies investigating plasma K levels in Down syndrome pregnancies may be worthwhile.

## Conclusions

In summary, this study showed the correlation between maternal plasma trace element levels and fetal NT thickness in the first trimester. The presence of essential elements tended to decrease NT thickness, while the presence of non-essential elements tended to increase this parameter. Maternal plasma K levels in the first trimester appeared to be associated with NT thickness. Further studies are needed to explore the mechanism by which maternal plasma K levels affect fetal NT thickness. Down syndrome screening using NT measurement may result in an increase in the false-negative rate if maternal plasma K levels are elevated. Hence, obstetricians should be aware of this when performing first trimester fetal screening.
